# Recent insights into the molecular mechanisms of the NLRP3 inflammasome activation

**DOI:** 10.12688/f1000research.8614.1

**Published:** 2016-06-22

**Authors:** Tomasz Próchnicki, Matthew S. Mangan, Eicke Latz

**Affiliations:** 1 Institute of Innate Immunity, University Hospital, University of Bonn, Bonn, Germany; 2 Department of Infectious Diseases and Immunology, University of Massachusetts Medical School, Worcester, MA, USA; 3 German Center for Neurodegenerative Diseases, Bonn, Germany; 4 Centre of Molecular Inflammation Research, Norwegian University of Science and Technology, Trondheim, Norway

**Keywords:** Inflammasome, pyroptosis, NLRP3, autoinflammatory disease

## Abstract

Inflammasomes are high-molecular-weight protein complexes that are formed in the cytosolic compartment in response to danger- or pathogen-associated molecular patterns. These complexes enable activation of an inflammatory protease caspase-1, leading to a cell death process called pyroptosis and to proteolytic cleavage and release of pro-inflammatory cytokines interleukin (IL)-1β and IL-18. Along with caspase-1, inflammasome components include an adaptor protein, ASC, and a sensor protein, which triggers the inflammasome assembly in response to a danger signal. The inflammasome sensor proteins are pattern recognition receptors belonging either to the NOD-like receptor (NLR) or to the AIM2-like receptor family. While the molecular agonists that induce inflammasome formation by AIM2 and by several other NLRs have been identified, it is not well understood how the NLR family member NLRP3 is activated. Given that NLRP3 activation is relevant to a range of human pathological conditions, significant attempts are being made to elucidate the molecular mechanism of this process. In this review, we summarize the current knowledge on the molecular events that lead to activation of the NLRP3 inflammasome in response to a range of K ^+^ efflux-inducing danger signals. We also comment on the reported involvement of cytosolic Ca ^2+^ fluxes on NLRP3 activation. We outline the recent advances in research on the physiological and pharmacological mechanisms of regulation of NLRP3 responses, and we point to several open questions regarding the current model of NLRP3 activation.

## Direct activation of NLRP3 by K ^+^ efflux

The stimulatory effect that cytosolic K ^+^ depletion has on IL-1β proteolytic processing and secretion from LPS-primed macrophages and monocytes, in response to compounds such as ATP or nigericin, was observed long before the discovery of inflammasomes ^1– 3^. This effect is now known to be mediated by NLRP3 ^4^, and K ^+^ efflux remains the best-characterized minimal stimulus for NLRP3 inflammasome activation ^5^. Conversely, incubation in media containing supraphysiological [K ^+^] can block NLRP3 inflammasome assembly in response to most of the identified NLRP3 triggers ^5^.

**Figure 1.**
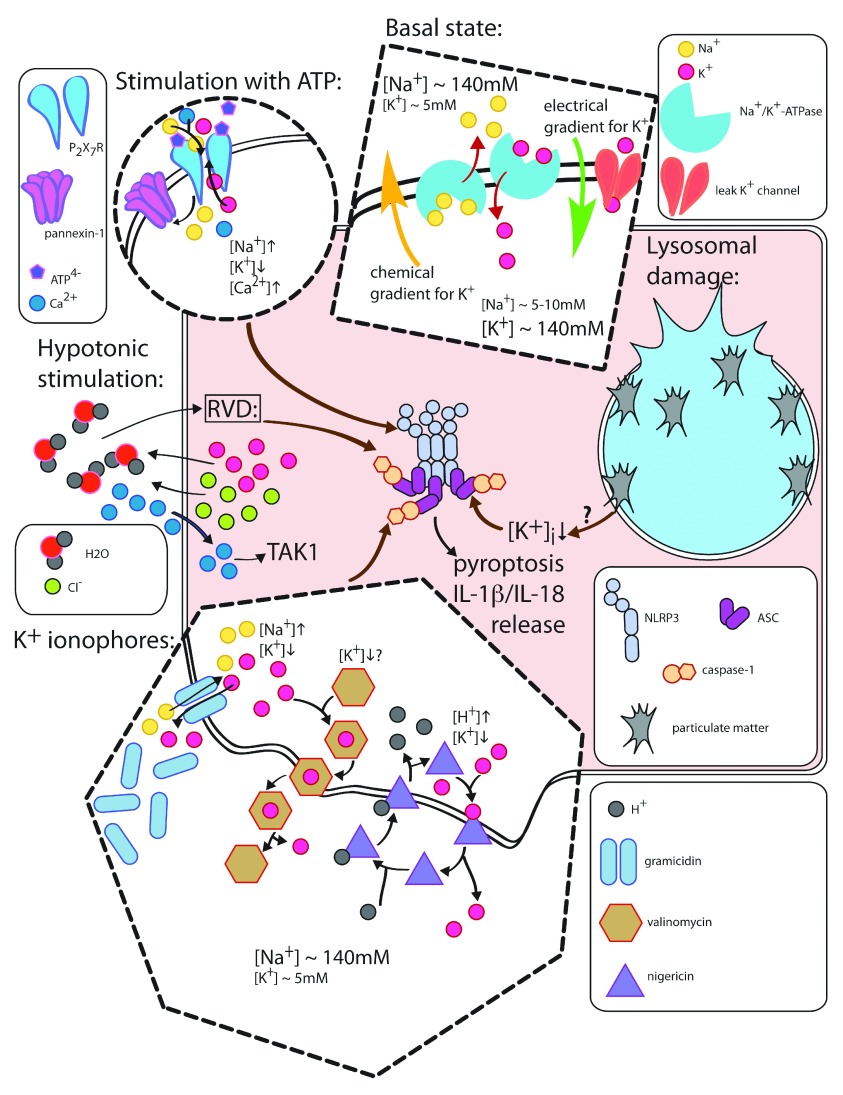
Canonical NLRP3 inflammasome activation by K ^+^ efflux Under basal conditions, high intracellular K ^+^ concentration is maintained by the activity of Na ^+^/K ^+^-ATPase, which actively imports K ^+^ ions into the cell and generates an electrical gradient that favors movement of cations into the cytoplasm. Together with leak K ^+^ channels, Na ^+^/K ^+^-ATPase contributes to the transmembrane potential, characterized by a slight excess of negative charges inside the cell. Under conditions of NLRP3 stimulation, this equilibrium is disturbed. ATP increases the open probability of P2X7R, a cation channel that allows for net exchange of intracellular K ^+^ ions for extracellular Na ^+^ or Ca ^2+^ ions. This produces a net K ^+^ efflux that acts as an NLRP3 activator. Activation of P2X7R is also accompanied by opening of pannexin-1 channels. During hypotonic stimulation, the regulatory volume decrease (RVD) response causes opening of K ^+^ and Cl ^-^ channels, driving an efflux of K ^+^ and Cl ^-^ ions to balance the intracellular and extracellular osmolarity values. To induce NLRP3 activation, this mechanism of K ^+^ ions depletion additionally requires an influx of Ca ^2+^ through TRP channels and activation of the kinase TAK1. NLRP3-activating K ^+^ ionophores produce a net K ^+^ efflux through different mechanisms. The peptide gramicidin can insert itself into plasma membranes, forming pores that are permeable to monovalent cations. This enables an exchange of intracellular K ^+^ for extracellular Na ^+^. Valinomycin, a neutral ionophore, is a cell-permeant compound that can bind to K ^+^ ions, replacing the hydration shell of this cation. Consequently, K ^+^ ions shielded by valinomycin molecules can pass across the plasma membrane without a requirement for opening a K ^+^-permeable pore. Nigericin is a carboxylic ionophore that can bind to H ^+^ or to K ^+^. Both the H ^+^- and K ^+^-bound forms of nigericin are plasma membrane permeant. In this way, nigericin mediates K ^+^ transport from the compartment with higher K ^+^ concentration to the compartment with lower K ^+^ concentration, concomitantly leading to a transient acidification of cytosol. In further stages, the increased cytosolic [H ^+^] can stimulate Na ^+^/H ^+^ exchangers to extrude H ^+^ ions from the cytosol, which is accompanied by Na ^+^ influx ^90^. Lysosomal damage caused by particulate materials or by other factors requires K ^+^ efflux to induce NLRP3 activation, but it is unknown which factors are involved in this K ^+^ depletion pathway.

The major classes of NLRP3 activators include extracellular ATP at millimolar concentrations, K ^+^ ionophores ^4^ and crystalline/particulate substances, or other factors that cause lysosomal destabilization ^6, 7^. All of these stimuli are known to decrease the cytosolic level of K ^+^ ions ^5^, but the mechanisms of K ^+^ efflux induction (summarized in Figure 1) differ for all classes of NLRP3 triggers. Before discussing these mechanisms in detail, it is important to reiterate some of the basic features of cellular K ^+^ homeostasis. Firstly, the cytosolic [K ^+^] ([K ^+^] _i_; ~140 mM) is much higher than the extracellular [K ^+^] ([K ^+^] _e_; ~5 mM), and this distribution is approximately reversed for Na ^+^ ions. This asymmetry is maintained by the Na ^+^/K ^+^-ATPase, an electrogenic ion pump transporting, during each cycle, two K ^+^ cations into the cytosol and three Na ^+^ cations into the extracellular milieu. Secondly, under basal conditions, the permeability of most mammalian plasma membranes is highest with respect to the K ^+^ cations and much lower for Na ^+^ and Ca ^2+^. Together, these factors contribute to sustaining the transmembrane potential of mammalian cells, which is characterized by a slight excess of negative charge on the inside of the cell. Each cycle of the Na ^+^/K ^+^-ATPase produces an electric charge difference of one elementary unit, and slow leakage of K ^+^ ions from the cell is not counterbalanced by a compensatory influx of another type of cation ^8^. Plasma membranes in basal states can generally be regarded as electrical insulators ^9^, so translocations of even small numbers of individual ions (much too small to cause any measurable changes in the intracellular concentration of the respective ion) produce significant changes in the value of transmembrane potential ^8^. Similarly, transporting ions in the direction opposite to the electrical gradient (i.e. cations to the outside of the cell or anions into the cell) requires significant energy input. In this light, the dramatic decrease in [K ^+^] _i_ required for NLRP3 activation, estimated as a drop of at least ~20–30% ^5^, can be expected to be accompanied by either a counter-flux of cations or a “co-flux” of anions, which should also be provided by NLRP3 stimuli.

Extracellular ATP at millimolar concentrations acts as an agonist of a ligand-gated cation channel called P2X7 receptor (P2X7R), which is permissive to K ^+^, Na ^+^, and Ca ^2+^ ^10^, allowing for cytosolic K ^+^ efflux balanced by the influx of extracellular Na ^+^ and Ca ^2+^. K ^+^ ionophores activating the NLRP3 inflammasome provide diverse pathways for K ^+^ transport. Gramicidin, a peptide ionophore, allows for K ^+^ efflux balanced by Na ^+^ influx by inserting into plasma membranes to form monovalent cation (Na ^+^/K ^+^)-permissive pores ^11^ in a manner electrochemically similar to the P2X7R. A different mechanism is employed by nigericin, a carboxylic ionophore that can exist in a free membrane-impermeant anionic form or as a neutral membrane-permeant complex when bound to a K ^+^ cation or a proton (H ^+^). In one electroneutral K ^+^ efflux cycle mediated by nigericin, the ionophore anion binds to H ^+^ on the outside of the cell, passes across the plasma membrane as nigericin-H, and releases the proton on the intracellular side. There, nigericin anion binds to K ^+^, which is subsequently transported across the plasma membrane as nigericin-K and released on the outside of the cell ^12^. This mechanism facilitates K ^+^ efflux by allowing an H ^+^ influx, leading to acidification of cytosol. Valinomycin, another NLRP3-activating K ^+^ ionophore, also forms equimolar complexes with K ^+^ but, unlike the neutral nigericin-K complexes, these complexes have a single positive charge (valinomycin-K ^+^) ^12^. Therefore, valinomycin-mediated K ^+^ efflux is electrogenic and can only occur until the chemical gradient that is pushing K ^+^ ions to the outside of the cell is balanced by the electric force drawing cations into the cell. It is currently unknown if such modest leakage of K ^+^ ions could be sufficient to activate NLRP3, or if the valinomycin-mediated K ^+^ efflux is accompanied by movement of another ionic species that would allow for a more pronounced decrease of [K ^+^] _i_.

It has been demonstrated that cell treatment with crystalline/particulate stimuli, representing pathophysiologically relevant NLRP3 activators, also leads to depletion of intracellular K ^+5^. However, the mechanism by which crystal-induced K ^+^ efflux occurs is currently not understood. It seems plausible that, during lysosomal rupture caused by crystals, mixing of lysosomal lumina (low [K ^+^], close to the extracellular concentration ^13^) with the cytosolic contents could passively decrease [K ^+^] _i_. However, the observation that the net K ^+^ content of cells decreases upon treatment with crystals ^5^ suggests, in the absence of data on the values of [K ^+^] _i_, that plasma membrane-resident K ^+^ channels or transporters might be involved in the crystal-elicited K ^+^ efflux. One model of NLRP3 activation by monosodium urate (MSU) crystals proposes that, upon phagocytosis, in the acidic lysosomal environment, Na ^+^ ions can be released from MSU particles leading to an increase in the osmolarity of the cell. This increase in osmolarity can be balanced by an influx of water from the extracellular space, which, it is suggested, dilutes [K ^+^] _i_ and thereby triggers NLRP3 activation ^14^. While the proposed mechanism explains how MSU crystal-induced lysosomal damage can activate the NLRP3 inflammasome, it cannot account for NLRP3 activation with stimuli such as silica or cholesterol crystals because these particles do not dissociate in the lysosomal pH and are consequently not expected to influence cellular osmolarity. Investigating the kinetics and molecular mechanism of K ^+^ loss in cells undergoing lysosomal damage may prove highly relevant for understanding how the different NLRP3 stimuli trigger inflammasome assembly and for designing treatments that specifically target NLRP3 activation by crystalline agents, which underlies multiple inflammatory diseases.

A number of other conditions that deplete cytosolic K ^+^ have been demonstrated to activate NLRP3. These include pharmacological inhibition of Na ^+^/K ^+^-ATPase ^3, 5^. Blocking Na ^+^/K ^+^-ATPase deprives cells of the mechanism maintaining both the asymmetric distribution of Na ^+^ and K ^+^ ions and the membrane potential, leading to a loss of K ^+^ ions. Under conditions of Na ^+^/K ^+^-ATPase inhibition, K ^+^ ions are no longer actively imported by the cell. Furthermore, dissipation of membrane potential, which accompanies Na ^+^/K ^+^-ATPase inhibition, eliminates the electrical force drawing K ^+^ ions into the cell. A similar scenario can be predicted in the case where cells are incubated in a K ^+^-free medium, which was also demonstrated to activate the NLRP3 inflammasome ^5^, because K ^+^-free media act as Na ^+^/K ^+^-ATPase inhibitors ^15^. Finally, a distinct mechanism of K ^+^ efflux is involved in NLRP3 activation by low-osmolarity media ^16^. Here, the regulatory volume decrease (RVD) response leads to a concerted efflux of K ^+^ and Cl ^-^ ions in an attempt to equilibrate intracellular and extracellular osmolarities ^17^. In this particular case, however, K ^+^ efflux does not seem to be sufficient for NLRP3 inflammasome activation, and an additional influx of Ca ^2+^ ions into the cytosol is required ^16^ (further discussed below).

## Molecular events occurring downstream of K ^+^ efflux

While depletion of intracellular K ^+^ is required for NLRP3 activation, little is known about how the change in [K ^+^] _i_ is sensed and how this information is further transduced to the inflammasome. Recently, an important development has helped to solve this question, as NEK7, a Ser/Thr kinase involved in mitotic cell division, has been identified as a factor specifically required for NLRP3 inflammasome activation downstream of K ^+^ efflux ^18– 20^. In response to NLRP3 activators, NEK7 is recruited to NLRP3 upstream of inflammasome formation (in a manner independent of ASC and caspases-1/11). NEK7 can also be detected in NLRP3/ASC specks, and formation of high-molecular-weight NLRP3 complexes that occurs upstream of ASC specking requires that NEK7 interacts with NLRP3. The NLRP3-NEK7 interaction is dependent on K ^+^ efflux and can be blocked by high [K ^+^] _e_^18^. Interestingly, the catalytic activity of NEK7 is required neither for its binding to NLRP3, nor for the activation of the NLRP3 inflammasome ^18, 20^.

The requirement for NEK7 in NLRP3 activation restricts this process to cells in interphase. At the endogenous level of NEK7, NLRP3 activators are able to enhance the interaction between NLRP3 and NEK7 in LPS-primed interphase cells but not in cells that have entered mitotic division, and LPS-primed interphase cells show a significantly higher level of caspase-1 activation than do their mitotic counterparts. Of note, NEK7 overexpression partially restores the responses to NLRP3 stimuli in mitotic cells, suggesting that the endogenous amount of NEK7 is not sufficient to simultaneously participate in both cell division and NLRP3 activation ^20^.

It remains unknown how NEK7 is recruited to NLRP3 in response to a decrease in [K ^+^] _i_ and whether elevating the interaction between NLRP3 and NEK7 above a certain threshold is sufficient to trigger the NLRP3 inflammasome assembly. In partial response to the first question, it was found that stimulation with ATP increases NEK7 phosphorylation ^20^. This increase could be blocked with N-acetylcysteine, a scavenger of reactive oxygen species (ROS) that also potently inhibits IL-1β release upon stimulation with ATP ^5, 20, 21^. However, it has not been clearly demonstrated that the enhanced phosphorylation of NEK7 is required for its interaction with NLRP3 and for NLRP3 inflammasome activation. Furthermore, shRNA-mediated silencing of NEK9, a kinase that interacts with NEK7 and causes its activation ^22^, does not inhibit NLRP3 activation ^18^, suggesting either that NEK7 activation is not required for the NLRP3 inflammasome assembly or that the ROS-dependent NEK7 activation occurs through an as-yet-unidentified mechanism. Further elucidation of this discrepancy and of the detailed mechanism by which NEK7 contributes to NLRP3 activation will be the next important step towards understanding the molecular mechanism of inflammasome assembly.

### Differential requirements for K ^+^ efflux and NEK7 presence in autoinflammatory disease-related NLRP3 mutants

Several single amino acid substitutions in NLRP3 are causative for systemic inflammation observed in a spectrum of autoinflammatory diseases known as cryopyrin-associated periodic syndromes (CAPS; cryopyrin being a synonym of NLRP3): neonatal onset multisystem inflammatory disease (NOMID), Muckle-Wells syndrome (MWS), and familial cold autoinflammatory syndrome (FCAS) ^23^. Of these, mainly the MWS-associated mutant NLRP3 ^R260W^ (whose mouse counterpart is Nlrp3 ^R258W^) has been studied with respect to the requirement for K ^+^ efflux for inflammasome assembly. Interestingly, activation of the Nlrp3 ^R258W^ mutant occurs in macrophages expressing Nlrp3 ^R258W^ in response to extracellular LPS stimulation (independent of any classical triggering stimuli) and without the requirement for K ^+^ efflux ^5^. However, NEK7 deficiency dramatically reduces the ability of Nlrp3 ^R258W^-expressing macrophages to activate the inflammasome in response to extracellular LPS ^18^. NLRP3 ^G775A^ and NLRP3 ^G775R^ mutants, which are mainly associated with NOMID, show a stronger association with NEK7 than does WT-NLRP3 when overexpressed in HEK293 cells and, conversely, the inflammasome activation-incompetent NLRP3 ^D946G^ mutant associates with NEK7 less strongly ^20^. Collectively, these observations suggest that some of the CAPS-causative mutations in NLRP3 could promote inflammasome activation by facilitating the interaction between NLRP3 and NEK7, but such a conclusion requires further elucidation of the mechanism by which NEK7 is involved in the activation of different NLRP3 variants.

### Non-canonical NLRP3 activation by caspase-11 involves K ^+^ efflux

Murine caspase-11 and its human orthologues caspases-4 and -5 are cytosolic LPS sensors ^24^ that, upon recognition of their ligand, trigger non-canonical inflammasome activation ^25^. This process consists of pyroptotic cell death that is independent of the canonical NLRP3 inflammasome components ^26^ and NLRP3-, ASC-, and caspase-1-dependent IL-1β/IL-18 processing and secretion ^25^. Recent studies demonstrated that NLRP3 activation downstream of caspase-11 is mediated by K ^+^ efflux ^27, 28^. This process is initiated by caspase-11-mediated cleavage of pannexin-1 ^26^, a plasma membrane-resident channel permeable to molecules and ions with a molecular weight of up to ~1 kDa ^29^. Two molecular events follow the proteolytic processing of pannexin-1: (a) K ^+^ efflux (a direct NLRP3 stimulus that induces mIL-1β secretion) and (b) release of ATP, which in turn acts as an agonist of P2X7R to promote cell death ^26^. Surprisingly, the levels of ATP released from cells upon caspase-11 activation and proposed to activate P2X7R are much lower (nanomolar concentrations) than the amounts of ATP typically required to activate this receptor when added as an exogenous stimulus ^30^. The mechanism by which intracellular LPS recognition increases macrophage sensitivity to extracellular ATP is not yet identified.

Another effector mechanism of caspase-11 activation involves proteolytic cleavage of a cytosolic protein, gasdermin D ^31– 33^. The signaling pathways activated upon cleavage of gasdermin D are unknown, but it was demonstrated that, while overexpression of full-length gasdermin D, or of gasdermin D C-terminal fragment, does not cause any apparent changes in cell physiology, expression of gasdermin D N-terminal fragment alone is highly cytotoxic ^32^. This observation evinces that the N-terminal fragment of gasdermin D is one of the downstream effectors of caspase-11. Important questions to be answered in further investigation of the role of gasdermin D in non-canonical inflammasome activation are whether overexpression of the gasdermin D N-terminal fragment is sufficient to activate the NLRP3 inflammasome and whether this process involves K ^+^ efflux. As gasdermin D N-terminal fragment alone is sufficient to cause pyroptosis, which is associated with plasma membrane disruption, it could be envisaged that this also leads to K ^+^ depletion.

**Figure 2.**
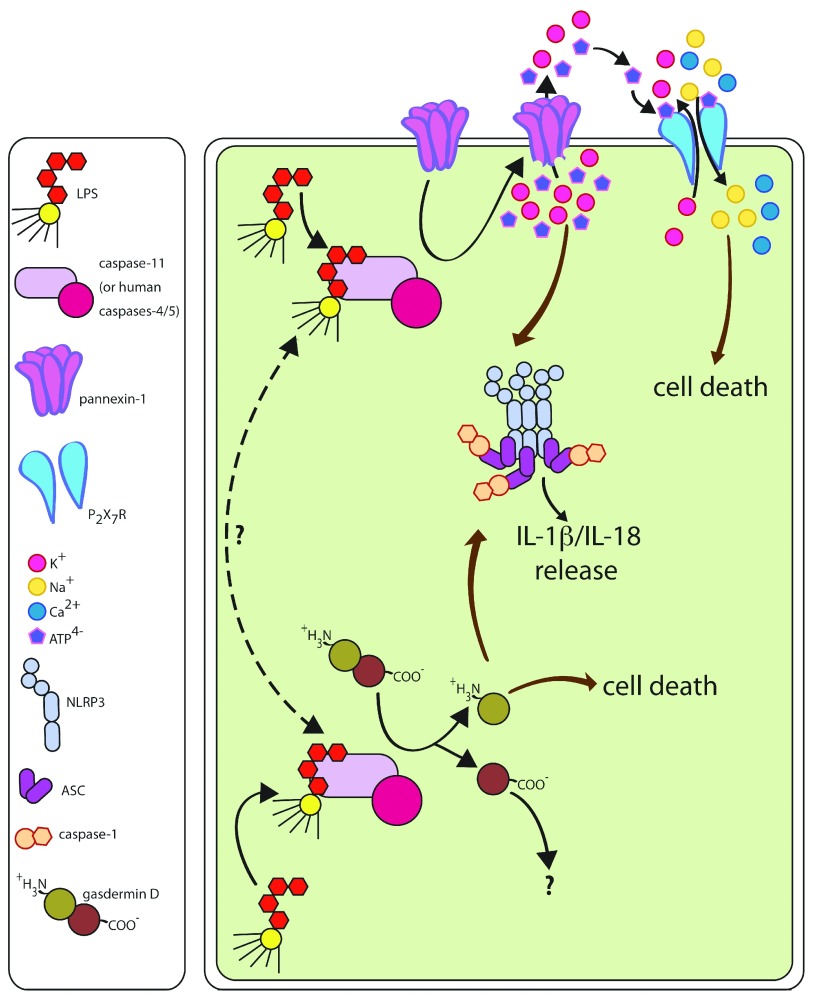
Noncanonical NLRP3 activation by cytosolic LPS. Upon recognition of LPS in the cytosol, caspase-11 cleaves pannexin-1 and gasdermin D. Cleavage of pannexin-1 leads to opening of the channel and leakage of K ^+^ and ATP from the cell into the extracellular space. This efflux of K ^+^ ions activates the NLRP3 inflammasome, causing proteolytic processing and secretion of IL-1β. Simultaneously, ATP acts as an agonist for the P2X7R, leading to NLRP3 inflammasome-independent pyroptotic cell death. Proteolytic cleavage of gasdermin D produces a highly toxic N-terminal fragment of this protein, which mediates both activation of the NLRP3 inflammasome (with subsequent IL-1β processing and secretion) and NLRP3-independent pyroptotic cell death. The relationship between two caspase-11 effectors, pannexin-1 and gasdermin D, is currently not understood.

Similar to pannexin-1 ^26^, gasdermin D is required for both cell death and IL-1β release in response to intracellular LPS ^32, 33^. While IL-1β secretion observed under conditions of intracellular LPS stimulation is dependent on NLRP3, pyroptosis elicited by intracellular LPS only depends on pannexin-1 ^26^ and gasdermin D ^32, 33^ and is unaffected in NLRP3-deficient cells. The recent discoveries on the caspase-11 effector mechanisms leading to non-canonical inflammasome activation and to pyroptosis are summarized in Figure 2. Future studies should address the questions of whether—and how—the caspase-11-mediated events (pannexin-1 and gasdermin D proteolytic processing) converge to orchestrate pyroptotic cell death. In particular, the mechanism by which gasdermin D N-terminal fragment induces cytotoxicity will have to be resolved, and the potential role of pannexin-1 cleavage in this process will have to be investigated more closely. Furthermore, there is a proposed mechanism by which the caspase-11/pannexin-1/NLRP3 axis triggers IL-1β/IL-18 secretion ^26^, but it remains unknown how gasdermin D is involved in this process.

Adding to our knowledge on canonical inflammasome activation, active caspase-1, alongside caspase-11, was also demonstrated to cleave gasdermin D ^32^. In response to activators of various canonical inflammasomes, gasdermin D-deficient macrophages exhibit delayed kinetics of cell death ^32^ and decreased levels of secreted IL-1β ^31– 33^, which suggests that caspase-1-catalysed proteolysis of gasdermin D is one of the effector mechanisms of pyroptosis and that it may contribute to non-classical cytokine secretion.

### Mechanisms of NLRP3 activation independent of K ^+^ efflux

Recently, it was reported that inhibition of glycolysis by targeting enzymes that catalyze two of the late reactions of this pathway, glyceraldehyde-3-phosphate dehydrogenase (GAPDH) or α-enolase, is sufficient to elicit inflammasome activation in an NLRP3-dependent manner ^34^. The proposed sequence of events consists of a decrease in cellular [NADH]/[NAD ^+^] ratio, and a consequent increase in the levels of mitochondrial ROS, which, it is suggested, are involved in NLRP3 activation ^35, 36^. Interestingly, supplementation with the glycolytic metabolite pyruvate (which is normally produced downstream of the inhibited steps of glycolytic cascade) or with succinate (one of the TCA cycle metabolites; both pyruvate and succinate can enhance TCA cycle activity) leads to a decrease in the level of both generated mitochondrial ROS and NLRP3 inflammasome activation ^34^. Such an observation suggests that in this pathway mitochondrial ROS act as NLRP3 stimuli. This mechanism of NLRP3 activation is uncommon because it does not require K ^+^ efflux: inhibition of GAPDH and α-enolase can trigger assembly of the NLRP3 inflammasome at supraphysiological [K ^+^] _e_^34^. Of note, NLRP3 activation under conditions of disrupted glycolytic flux could have profound pathophysiological significance as, for example, macrophages infected with *Salmonella typhimurium* exhibit decreased levels of NADH ^34^. Given that infection with *S. typhimurium* is an activator of the NLRP3 inflammasome ^37^ and that *S. typhimurium*-mediated NLRP3 activation can be abrogated by pyruvate supplementation ^34^, this metabolic signature may constitute an important signal in inflammasome activation.

Of note, it has been demonstrated that efficient glycolysis is required for NLRP3 activation by canonical K ^+^-depleting stimuli ^38^. Furthermore, the metabolic changes resulting from inhibition of glycolysis were not observed in cells treated with nigericin, a canonical K ^+^ efflux-dependent NLRP3 activator ^34^, suggesting that the newly discovered pathway is a distinct mechanism of NLRP3 activation rather than simply an event occurring downstream of cellular K ^+^ depletion. In further support of this conclusion, canonical activation of the NLRP3 inflammasome with stimuli such as nigericin or ATP cannot be inhibited by supplementation with pyruvate ^34^. It remains unknown whether the NLRP3 inflammasome assembly in response to glycolysis inhibitors relies on a NEK7-dependent mechanism. However, this is unlikely in light of the observations that (a) the interaction between NEK7 and NLRP3 requires K ^+^ efflux and (b) the K ^+^/H ^+^ ionophore nigericin does not inflict metabolic changes resembling those caused by inhibition of glycolysis.

Another mechanism of NLRP3 activation independent of K ^+^ efflux is observed in monocytes but is restricted to several species (e.g. humans or pigs) and not observed in murine cells. For this mechanism of NLRP3 activation, termed “alternative inflammasome activation”, extracellular LPS is a stimulus sufficient to elicit mature IL-1β release but not to cause ASC speck formation or pyroptosis. LPS acts as an agonist of TLR4, leading to engagement of the adaptor protein TRIF and of the RIPK1-FADD-caspase-8 signaling cascade, culminating in caspase-1 activation in an NLRP3- and ASC-dependent manner ^39^. The specific nature of the signaling events that drive alternative NLRP3 activation as well as the fact that this mechanism does not lead to the generation of ASC specks may collectively suggest an involvement of a distinct, K ^+^ efflux-independent active NLRP3 conformation in this process.

## Ca ^2+^ influx is not sufficient, and may not be required, for NLRP3 activation

Based on the ability of certain NLRP3 stimuli to increase cytosolic [Ca ^2+^] ([Ca ^2+^] _i_), and on the inhibitory effect that several small-molecule compounds targeting intracellular Ca ^2+^ have on NLRP3 activation, it was proposed that [Ca ^2+^] _i_ ions could be involved in NLRP3 activation ^40– 42^. The major mechanisms of [Ca ^2+^] _i_ increase in the cytosol are (a) Ca ^2+^ influx from the lumen of endoplasmic reticulum (ER) through a ligand-gated ion channel called inositol trisphosphate (IP _3_) receptor (IP _3_R), a downstream effector of the phospholipase C (PLC) family, and (b) entry of extracellular Ca ^2+^ ions through plasma membrane-resident Ca ^2+^ channels ^43^. Important Ca ^2+^-buffering organelles are (c) mitochondria, which can either absorb or release Ca ^2+^ under different conditions ^44^. All of these pathways have been implicated in the activation of NLRP3. In favor of the hypothesis that the ER-derived Ca ^2+^ ions could be a stimulus of NLRP3, a range of small-molecule IP _3_R antagonists and PLC inhibitors have been consistently demonstrated to inhibit activation of the NLRP3 inflammasome ^40– 42^. However, the observed levels of inhibition vary between the different studies, and in some cases the applied concentrations of small-molecule compounds required to inhibit NLRP3 significantly surpass their IC _50_ values reported for other processes ^45, 46^. Furthermore, artificially increasing [Ca ^2+^] _i_ with thapsigargin, an inhibitor of the sarcoplasmic/endoplasmic reticulum Ca ^2+^-ATPase (SERCA; an ion pump transporting Ca ^2+^ from the cytosol into the ER lumen, responsible for maintaining the steep [Ca ^2+^] gradient between the ER lumen and the cytosol) either inhibits ^40^ or does not influence ^47^ NLRP3 activation, demonstrating that translocation of Ca ^2+^ ions into the cytosol is not sufficient to trigger that process. Of note, thapsigargin was demonstrated to elicit modest IL-1β secretion from LPS-primed human macrophages, but it is not known whether the mature form of the cytokine is secreted and whether this process is mediated by NLRP3 ^48^. Furthermore, thapsigargin was demonstrated to cause NLRP3 activation by inducing ER stress, but the role of Ca ^2+^ in this process has not been studied ^49^.

The current evidence for the involvement of extracellular Ca ^2+^ in the activation of NLRP3 strongly suggests that this pool of Ca ^2+^ does not play a role in the inflammasome assembly. Supporting this is the observation that all tested canonical NLRP3 stimuli that act by depleting cytosolic K ^+^ can activate the inflammasome in Ca ^2+^-free extracellular buffers ^47, 50^. In several studies, a contradictory effect of extracellular Ca ^2+^ depletion was reported ^40, 48^ but, in some cases at least, such observations may have resulted from simultaneous application of BAPTA-AM and Ca ^2+^-free buffer ^48^, which can be expected to interfere with Ca ^2+^ fluxes deriving from a range of different sources. Nevertheless, there is currently no convincing explanation as to why in certain experimental systems the removal of extracellular Ca ^2+^ seems to inhibit NLRP3, while in other setups such treatment does not interfere with NLRP3 activation. A second argument supporting the claim that extracellular Ca ^2+^ is not required for the NLRP3 inflammasome activation comes from the observation that K ^+^ ionophores elicit NLRP3 inflammasome assembly in the absence of any significant changes in [Ca ^2+^] _i_^47^. Surprisingly, one study reports that human macrophages can secrete IL-1β in response to ionomycin, a Ca ^2+^ ionophore, but, similar to the case of thapsigargin, the involvement of NLRP3 is not proven and it is not demonstrated that the released form of the cytokine is proteolytically processed ^48^.

Interestingly, in the NLRP3 response to hypotonic environments, extracellular Ca ^2+^ influx through mechanosensitive TRP channels and consequent activation of the kinase TAK1 were both demonstrated to be required for activation of the inflammasome alongside RVD-mediated K ^+^ efflux ^16^. It is unknown whether this mechanism also contributes to inflammasome assembly by all NLRP3 stimuli, but it is suggested that TAK1 is involved in NLRP3 activation induced by lysosomal damage ^51^. Finally, one study proposed that Ca ^2+^ influx into the cytosol follows K ^+^ efflux caused by NLRP3 stimuli and that this Ca ^2+^ flux promotes activation of the inflammasome by enhancing mitochondrial ROS generation ^52^. However, there are two major limitations to this conclusion. First, the only method applied for targeting cytosolic Ca ^2+^ was cell loading with BAPTA-AM, which may exert numerous off-target effects ^53^. Secondly, the only tested NLRP3 activator was ATP, a cation channel opener and inducer of PLC (through interaction with P2Y2 receptor ^54^), which makes it challenging to dissect the relative contributions of the various ion fluxes in the process of inflammasome activation.

The mitochondrial Ca ^2+^ stores are the most difficult to perturb experimentally, and consequently their potential role in NLRP3 activation is not well understood. Several reports suggest that an increased Ca ^2+^ uptake by the mitochondria may promote mitochondrial damage and NLRP3 responses ^40, 55, 56^, although it was also demonstrated that mitochondrial damage inflicted by canonical NLRP3 activators is at least in part dependent on NLRP3 and caspase-1 ^57^. The transporter responsible for mitochondrial Ca ^2+^ uptake during NLRP3 activation by the membrane attack complex (a component of the complement cascade) and by *Pseudomonas aeruginosa* has been identified as mitochondrial Ca ^2+^ uniporter (MCU) ^55, 56^. The specific factors that could tie an increase in mitochondrial [Ca ^2+^] to NLRP3 inflammasome assembly have not been identified, and it remains unknown whether MCU is involved in NLRP3 responses to its classical, better-characterized activators.

The collective evidence regarding the role of Ca ^2+^ in the activation of NLRP3 suggests that elevation in [Ca ^2+^] _i_ is not required for the assembly of this inflammasome. However, a modulatory role for this ion cannot be excluded, especially in light of two puzzling observations: (a) inhibition of NLRP3 responses by BAPTA-AM ^41, 47^ and (b) inhibition of NLRP3 activation by siRNA knock-down of a G _q_α-coupled G-protein-coupled receptor (GPCR) called Ca ^2+^-sensing receptor (CaSR) ^42^. Given that BAPTA-AM has off-target effects apart from scavenging Ca ^2+^ ions inside the cell ^53^ and that Ca ^2+^ ions are not the only ligand of CaSR ^58^, re-evaluation of the mechanisms by which application of BAPTA-AM or suppression of CaSR signaling interfere with NLRP3 activation could provide valuable insights into the molecular events that regulate NLRP3 inflammasome assembly. We further discuss some aspects of GPCR/CaSR signaling below.

## Physiological and pharmacological modulation of NLRP3

The relevance of NLRP3 in human pathologies has led to research regarding both the intrinsic mechanisms that limit inflammasome activation and the possibility of pharmacological targeting of NLRP3. Even though such studies are challenging, because it is unclear how the K ^+^ efflux is transduced to NLRP3, they have nevertheless resulted in discoveries that processes such as cAMP signaling and autophagy can interfere with NLRP3 activation and in identification of several classes of exogenous small-molecule compounds that can act as specific inhibitors of NLRP3 activation.

### Inhibition of the NLRP3 inflammasome by cAMP

In recent years, the interest in how GPCRs could regulate NLRP3 responses resulted in an observation that increasing [cAMP] _i_ inhibits the activation of the NLRP3 inflammasome ^42^. Specifically, treating cells with pharmacological activators of adenylyl cyclases ^42^, or with agonists of GPCRs that enhance adenylyl cyclase activity ^59, 60^, leads to a decrease in NLRP3 activation in response to classical NLRP3 stimuli. Conversely, NLRP3 stimuli were demonstrated to decrease [cAMP] _i_^42^, although it is currently not clear whether this decrease occurs upstream or downstream of NLRP3 activation. One study suggested that inhibition of adenylyl cyclase enzymatic activity (surprisingly, using KH7, an inhibitor targeting the GPCR-independent soluble adenylyl cyclase and not acting on the GPCR-regulated transmembrane adenylyl cyclases ^61^) might be sufficient to activate the NLRP3 inflammasome ^42^, but this result could not be reproduced, possibly due to differences in the applied concentrations of the compound ^60^. Of note, inhibitors of transmembrane adenylyl cyclases also do not act as NLRP3 inflammasome activators, pointing to a modulatory role of [cAMP] _i_ rather than its decrease being the direct NLRP3 stimulus.

Pharmacological targeting of various cAMP-binding proteins that act as downstream effectors of adenylyl cyclase activation revealed that the inhibitory effect that cAMP exerts on NLRP3 activation cannot be ascribed to the currently known cAMP targets ^42, 59, 60^. In the study that identified cAMP as a regulator of NLRP3, it was proposed that NLRP3 could form a complex with cAMP ^42^, and recently it was demonstrated that the NLRP3-cAMP complex recruits ubiquitin ligase MARCH7, which in turn labels NLRP3 for degradation in autophagosomes ^60^. It is suggested that this process down-regulates NLRP3 signaling. Nevertheless, there are still open questions about the mechanism of inhibition of NLRP3 responses by cAMP. This model suggests a direct interaction between cAMP and NLRP3, in which the nucleotide-binding domain (NBD) of NLRP3 is involved ^42^. However, sequence analysis of NLRP3-NBD does not suggest the presence of a cyclic nucleotide-binding fold along with the ATP-binding site ^62^. Furthermore, even if the cAMP-binding and ATP-binding sites were in fact the same structural interface, it is not likely that cAMP could compete with ATP for binding to NLRP3, given that in living cells [ATP] _i_^63, 64^ is much higher than [cAMP] _i_^65^. Another problematic aspect of studies on the influence of cAMP on activation of NLRP3 is the consistent use of KH7 ^59, 60^, the inhibitor of soluble, GPCR-independent adenylyl cyclases ^66^, to interfere with events that, it is proposed, occur downstream of GPCR-responsive transmembrane adenylyl cyclases. Applying genetic rather than pharmacological approaches to the studies on the influence of cAMP on NLRP3 activation and a more thorough investigation of the roles of established cAMP-binding proteins in this process could potentially provide a greater insight into the mechanism of NLRP3 response inhibition by cAMP.

### Regulation of inflammasome responses by autophagy

**Figure 3.**
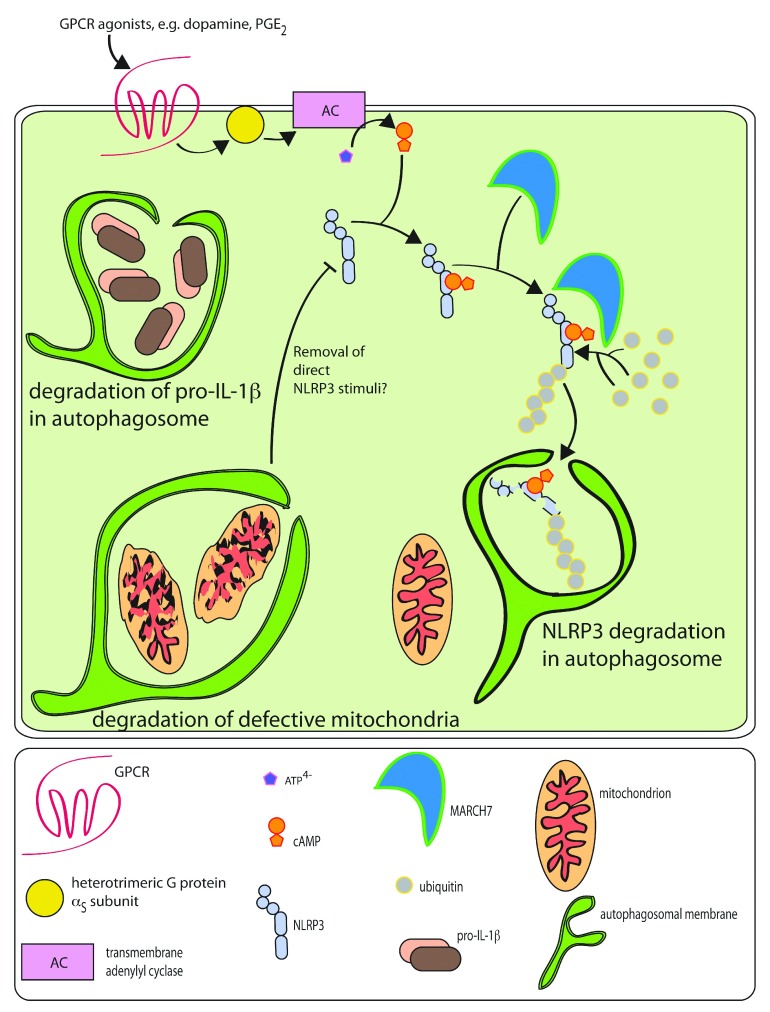
Modulation of the NLRP3 inflammasome by cAMP and autophagy Several physiological mechanisms regulate NLRP3 responses on the cellular level. Agonists of G _S_-coupled GPCRs stimulate the generation of cAMP by transmembrane adenylyl cyclases. cAMP is believed to bind to the nucleotide-binding domain of NLRP3. This formed NLRP3-cAMP complex recruits the ubiquitin ligase MARCH7 that polyubiquitinates NLRP3, targeting it for autophagosomal degradation. Autophagosomes are also the organelles responsible for degradation of pro-IL-1β (the inactive pro-form of the proinflammatory cytokine IL-1β), which is a more general mechanism controlling the inflammatory responses mediated by a range of inflammasomes. Finally, mitophagy is a way to dispose of damaged mitochondria that starts with sequestering them in autophagosomes. Autophagosomal degradation of dysfunctional mitochondria curbs the inflammasome responses, possibly by removing the source of direct NLRP3 activators.

Autophagy is emerging as a central process regulating multiple inflammasome responses at several levels. Pro-IL-1β can be degraded in autophagosomes, leading to decreased inflammatory responses to a range of stimuli ^67^. In addition, it is also proposed that autophagy specifically controls NLRP3 activation over other characterized inflammasomes. Suppression of the autophagic processes impairs homeostatic turnover of mitochondria, promoting mitochondrial damage that contributes to caspase-1 activation in response to ATP ^68^, as well as in the NLRP3 response to influenza A virus infection ^69^. A decrease in the number of autophagosomes was also reported in response to palmitate, a long-chain fatty acid previously demonstrated to activate NLRP3 ^70^. However, activation of NLRP3 using nigericin or crystalline stimuli enhanced autophagy, ^71^ targeting inflammasome components for degradation in the autophagosomes. Collectively, these observations suggest that reduction in the autophagic processing of cellular contents may support NLRP3 inflammasome responses. Conversely, increased autophagy may act as a regulator of the NLRP3 inflammasome specifically and a regulator of IL-1β-based inflammation generally by a negative feedback loop. The recent discoveries that dopamine decreases cellular responses to NLRP3 activators by targeting this inflammasome sensor protein for degradation in autophagosomes ^60^ and that NF-κB signaling can inhibit activation of NLRP3 by stimulating the autophagic turnover of dysfunctional mitochondria ^72^ demonstrate that this regulatory mechanism can position immune cells towards a state of decreased sensitivity to NLRP3 stimuli even before they encounter inflammasome activators. The proposed mechanisms of regulation of NLRP3 responses by autophagy and by cAMP (discussed earlier) are summarized in Figure 3.

### Inhibition of the NLRP3 inflammasome by compounds containing a sulfonylurea moiety

The first observation that compounds containing a sulfonylurea moiety potently inhibit ATP- or hypotonicity-induced IL-1β processing and release predates the discovery of inflammasomes ^73^. This phenomenon was later recognized as specific inhibition of the NLRP3 inflammasome ^74^, and until now virtually all validated NLRP3 activators are sensitive to sulfonylurea-containing compounds, such as glyburide or CP-456,773 ^75^. Sulfonylurea drugs seem to specifically inhibit the triggering step of NLRP3 activation without affecting the NF-κB signaling-related priming step or the activation of other inflammasomes ^74, 75^. Compounds containing sulfonylurea moieties have been tested, as NLRP3 inhibitors, in several animal inflammatory disease models, usually with encouraging results ^75– 79^. Of note, alternative inflammasome activation (described in more detail above) can also be blocked by CP-456,773 ^39^.

The mechanism by which sulfonylurea compounds inhibit NLRP3 activation is currently not understood. Given that an important target of these pharmaceuticals are K ^+^ channels ^80– 82^ and that K ^+^ efflux is required for NLRP3 activation ^5^, one concept would be that sulfonylureas could impede K ^+^ efflux from cells treated with NLRP3 stimuli. However, not all sulfonylurea drugs can inhibit inflammasome activation ^74^ and, conversely, sulfonylurea compounds were demonstrated not to prevent K ^+^ efflux caused by NLRP3 activators ^75^, which collectively suggests that these inhibitors act downstream of K ^+^ depletion and that the inhibition mechanism is not related to the activity of these compounds on K ^+^ channels. Glyburide was shown to inhibit the ATPase activity of NLRP3, but it is unclear whether other drugs from this class can act in a similar manner, and if this observation is related to the glyburide-mediated inhibition of inflammasome formation. CP-456,773 (CRID3 ^83^, which has recently been renamed to MCC950 ^75^) has been demonstrated not to affect the Ca ^2+^ flux in cells treated with ATP ^75^, which, some studies suggest, plays a role in NLRP3 activation ^40^. The influence of CP-456,773 on other molecular events connected to NLRP3 activation, such as the production of ROS, decrease in [cAMP] _i_, or recruitment of NEK7 to NLRP3, has not yet been tested.

Attempts to identify the molecular target of CP-456,773 showed that this compound interacts with proteins from the glutathione S-transferase family ^83^, but so far none of these have been shown to transduce the information about K ^+^ efflux to the NLRP3 inflammasome. There is conflicting evidence regarding the ability of sulfonylurea drugs to inhibit the activation of CAPS-related NLRP3 mutants, as glyburide has been shown not to affect IL-1β release from cultured monocytes from an FCAS-affected patient ^74^, but CP-456,773 suppressed mutant NLRP3 activation in both the mouse model of MWS and in monocytes from an MWS-affected patient ^75^. The NLRP3 mutants investigated in these studies had different amino acid substitutions, which, together with other differences in the experimental systems, could have led to this apparent discrepancy. A more comprehensive study addressing the sensitivity of a range of hyperactive NLRP3 mutants to sulfonylurea compounds could provide more insight into whether these drugs can inhibit inflammasome activation by these protein variants and what the mechanism of inhibition could be.

### Inhibition of the NLRP3 inflammasome by cysteine-modifying compounds

Several recent studies demonstrated that NLRP3 activation can be abolished by pre-treatment of cells with various compounds that contain a Michael acceptor group (a double C=C bond in the α position with respect to a carbonyl [-C=O] or a nitro group [-NO _2_]) ^84, 85^. In biological systems, these compounds can covalently modify protein Cys residues that are not engaged in disulfide bond formation ^86, 87^. This mechanism explains the inhibition of the NLRP3 inflammasome both by compounds that are generally regarded as NLRP3 inhibitors (e.g. parthenolide and BAY 11-7082) ^85^ and compounds whose ability to inhibit NLRP3 activation has been identified as an “off-target” effect (e.g. the Syk kinase inhibitor 3,4-methylenedioxy-β-nitrostyrene [MNS]) ^84^. Importantly, the issue of specificity of the tested inflammasome inhibitors has also been addressed in the cited studies and, while MNS and BAY 11-7082 have been demonstrated to selectively inhibit NLRP3, parthenolide was also able to block other inflammasome responses ^84, 85^. This observation is probably related to parthenolide-mediated direct inhibition of caspase-1 ^85^ (which contains a Cys residue that is essential for its catalytic activity and which can also be modified by Michael acceptors ^87^).

In further investigation of the mechanism of NLRP3 inhibition by Cys-modifying compounds, two consecutive structure-activity relationship studies demonstrated that Michael acceptors with very diverse chemical structures can interfere with NLRP3 activation (assessed by IL-1β release and pyroptotic LDH release) ^88, 89^. Of note, these compounds are capable of inhibiting NLRP3 ATPase activity ^88, 89^. Furthermore, several Michael acceptors moderately but significantly inhibit the activation of CAPS-related NLRP3 variants, but their potency on these NLRP3 mutants is lower compared to the inhibitory influence exerted on WT-NLRP3 ^89^.

When applying compounds that contain a Michael acceptor moiety to investigate the molecular mechanism of NLRP3 activation, several issues have to be considered. First, the downstream effector of NLRP3 is caspase-1, a Cys protease whose catalytic activity depends on an unmodified, free Cys residue. This implies that Michael acceptors, at high enough concentrations, may obscure various experimental readouts that rely on caspase-1 activity, such as assessing inflammasome speck formation using the caspase-1-targeting FLICA reagent, IL-1β/IL-18 release, or pyroptotic LDH release, even if the particular compounds-of-interest do not directly interfere with NLRP3 activation. Second, the ability of Michael acceptors to directly interact with NLRP3/modify Cys residues in NLRP3 ^84, 89^ only suggests, but does not prove, that the NLRP3-Cys modification constitutes the mechanism of inflammasome inhibition by these compounds. Further insights into the structural basis of NLRP3 activation and into the possible influence of Michael acceptors on that process are required to resolve this issue.

## Conclusions and future directions

In recent years, a number of molecular players involved in NLRP3 activation have been identified. Most importantly, the direct interaction of NLRP3 with the kinase NEK7 has been described, and its importance for the assembly of the NLRP3 inflammasome has been demonstrated. Major progress has also been made in our understanding of how intracellular LPS triggers caspase-11, leading to proteolytic processing of pannexin-1 and gasdermin D, and to non-canonical NLRP3 inflammasome activation. Finally, the mechanism by which human monocytes activate NLRP3 in response to extracellular LPS as a single stimulus has been solved and shown to rely on TLR4/TRIF-mediated activation of the RIPK1-FADD-caspase-8 cascade. On the other hand, physiologically relevant mechanisms, such as autophagy and cAMP signaling, have been proposed to down-regulate the activation of NLRP3, demonstrating the physiological importance of limiting NLRP3 inflammasome responses.

However, there still remain unanswered questions about the molecular events that link cytosolic K ^+^ depletion to NLRP3-/NEK7-dependent inflammasome formation. Furthermore, the discovery of the dual function of gasdermin D (as a downstream effector of caspase-11 required for non-canonical NLRP3 activation and a substrate of inflammatory caspases required for pyroptosis) calls for closer investigation of the mechanism of action of this protein. In light of the reported K ^+^ efflux-independent modes of NLRP3 triggering that include alternative NLRP3 inflammasome activation and NLRP3 inflammasome activation upon inhibition of glycolysis, the relative contributions of these pathways to inflammatory responses will have to be evaluated. Finally, the mechanisms by which compounds containing sulfonylurea or Michael acceptor moieties cause NLRP3 inhibition will have to be defined, which may open new possibilities for potential future therapeutic applications of these molecules.
